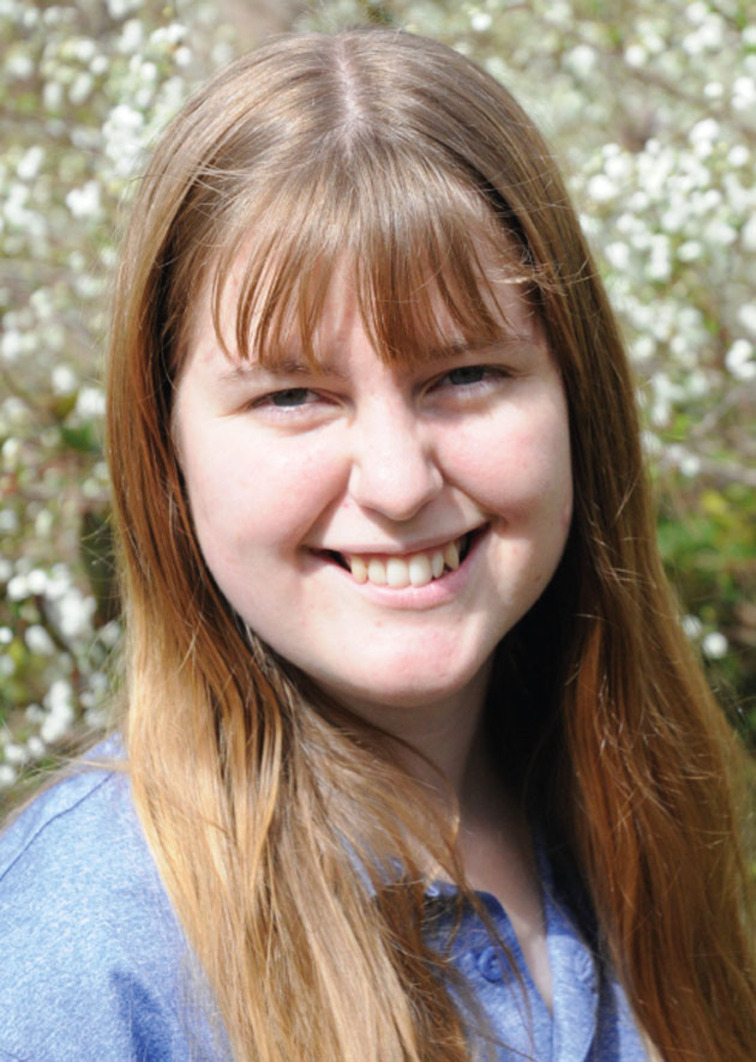# Regioselective Deacetylation in Nucleosides and Derivatives

**DOI:** 10.1002/cbic.202400360

**Published:** 2024-09-05

**Authors:** Charis Grabbe, Li Cai

**Affiliations:** ^1^ Department of Chemistry University of South Carolina Lancaster 476 Hubbard Dr Lancaster, SC 29720 USA

**Keywords:** Chemoselectivity, Deacetylation, Enzyme catalysis, Nucleosides, Regioselectivity

## Abstract

Nucleoside analogues are a promising class of natural compounds in the pharmaceutical industry, and many antiviral, antibacterial and anticancer drugs have been created through structural modification of nucleosides scaffold. Acyl protecting groups, especially the acetyl group, play an important role in the protection of hydroxy groups in nucleoside synthesis and modification; consequently, numerous methodologies have been put forth for the acetylation of free nucleosides. However, for nucleosides that contain different *O*‐ and *N*‐based functionalities, selective deprotection of the acetyl group(s) in nucleosides has been studied little, despite its practical significance in simplifying the preparation of partially or differentially substituted nucleoside intermediates. In this mini‐review, recent approaches for regioselective deacetylation in acetylated nucleosides and their analogues are summarized and evaluated. Different regioselectivities (primary ester, secondary ester, full de‐*O*‐acetylation, and de‐*N*‐acetylation) are summarized and discussed in each section.

## Graphical abstract

Selective deprotection of the acetyl group(s) in acetylated nucleosides demonstrates practical significance in synthetic processes. It simplifies the preparation of partially or differentially substituted nucleoside intermediates. In this mini‐review, we summarized and evaluated recent approaches for regioselective deacetylation in acetylated nucleosides and their analogues. Different regioselectivities (primary ester, secondary ester, full de‐*O*‐acetylation, and de‐*N*‐acetylation) are summarized and discussed within each section of this report.

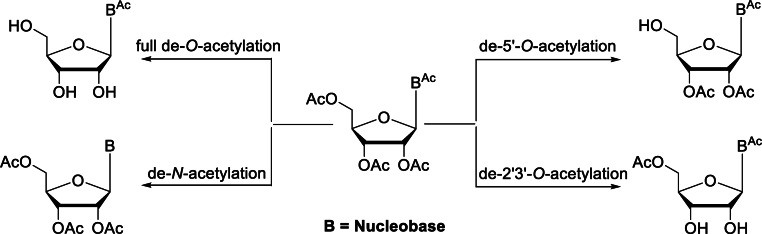

## 1 Introduction

Nucleosides, oligonucleotides, and their modified derivatives have been widely studied for therapeutic and diagnostic purposes in recent years.[^1^, ^2^, ^3^, ^4^, ^5^] They are starting materials and key building blocks for the synthesis of various types of RNAs and DNAs.[^6^, ^7^] Nucleoside derivatives have also been widely used as antiviral drugs[^8^, ^9^, ^10^, ^11^] against several viruses, including ebolavirus, hepatitis C virus, HIV, MERS, SARS‐CoV, and particularly the coronavirus (COVID‐19), and they show promise as anticancer and cardioprotective agents.[^12^, ^13^, ^14^, ^15^, ^16^, ^17^]

**Figure 1:**
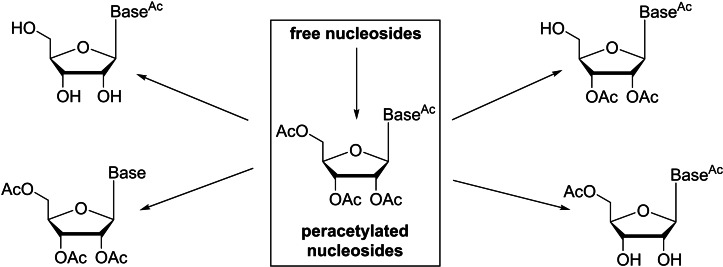
Partially or differentially acetylated nucleosides derived from peracetylated nucleosides.

Direct modification of free nucleosides is often inhibited, as the nucleophilic and electrophilic sites (2′, 3′, 5′‐OH and nucleobase ‐NH_2_) in nucleosides are linked with structural functionalization.[^18^, ^19^, ^20^, ^21^, ^22^, ^23^, ^24^] Therefore, orthogonal protection is necessary to achieve partially or differentially substituted nucleoside intermediates, and selective deprotecting strategies are strongly desired in nucleoside chemistry. Esters, especially the acetyl ester, play an important role in the protection of the hydroxy (and amino) groups in organic synthesis because acetylation of hydroxy groups is generally a simple and low‐cost step.[^25^, ^26^, ^27^] Consequently, numerous methodologies have been put forth for the acetylation of free nucleosides.[^20^, ^26^, ^28^, ^29^] In addition, acetylation often enhances the bioavailability and cell membrane permeability of nucleosides and carbohydrates, which are crucial for their applications in current chemical glycobiology.[^30^, ^31^, ^32^, ^33^] Despite the high efficiency of peracetylation reactions (generally nearly quantitative),[^34^, ^35^] selective deacetylation in peracetylated nucleosides has been much less studied. Thus, partially or differentially acetylated nucleosides for structural functionalization are still commonly prepared via a multi‐step protection and deprotection strategy, which requires tedious chromatographic purifications. In this mini‐review, we considered the practical significance of the shorter peracetylation‐selective deacetylation reaction sequence (Figure 1) and summarized and evaluated approaches for regioselective deacetylation in acetylated nucleosides and their analogues. To the best of our knowledge, this is the first time these reactions have been summarized and reviewed. Within each section of this manuscript, different regioselectivities (primary ester, secondary ester, full de‐*O*‐acetylation, and de‐*N*‐acetylation) were discussed (Figure 1).

## 2 Selective De*‐O‐*Acetylation – Primary Ester

**Scheme 1:**
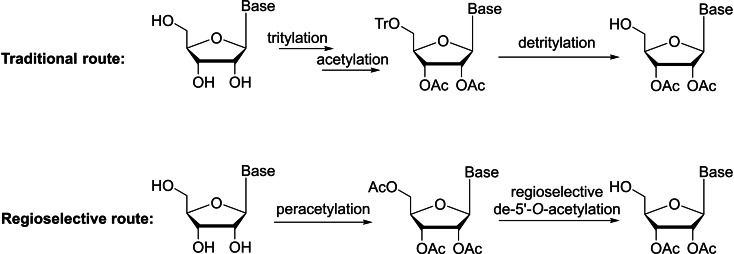
Routes for the synthesis of acetylated nucleosides with a free 5′‐OH.

Protected nucleosides with a free 5′‐OH function are critical in preparing the key intermediates for many biologically active molecules, such as nucleotides[^36^, ^37^, ^38^, ^39^] and modified nucleoside analogues.^[40]^ The primary 5′‐OH is the least sterically hindered hydroxy group of nucleosides and shows the highest nucleophilicity in reactions.^[20]^ Traditionally, acetylated nucleosides with a free 5′‐OH are prepared using a three‐step protection and deprotection strategy[^20^, ^41^] via a temporary protecting group, such as the acid‐labile trityl and *p*‐methoxy‐substituted trityl protecting groups, at the 5′ position (Scheme 1, traditional route). The process also requires tedious chromatographic purifications.^[42]^ Considering the nearly quantitative transformation for the peracetylation reaction,[^29^, ^43^] regioselective cleavage of the primary acetyl ester on fully acetylated nucleosides is a much more straightforward and efficient route (Scheme 1, regioselective route). In addition, reactive groups in the ribose or in the base may interfere, and thus a mild deacetylation procedure is usually of great synthetic value.

Chemical methods for selective cleavage of the primary acetyl ester on fully acetylated nucleosides are very limited. One of the most notable methods uses a neutral organotin catalyst, [*t*Bu_2_SnOH(Cl)]_2_, in methanol.[^34^, ^44^, ^45^, ^46^] A variety of functional groups, such as ether, acetal and alkyne, were well‐tolerated in the deacetylation reaction due to its mild reaction conditions and the fact that the reaction can be run in the presence of co‐solvent.^[34]^ Because acetyl groups from secondary and tertiary alcohols were less reactive under catalysis, and competition reactions showed that acetyl groups from primary alcohols were preferentially or exclusively deprotected in the presence of a secondary or tertiary alcohol derivative, this reagent could be perfectly applied in carbohydrate/nucleoside chemistry.^[47]^ Deprotection of the primary acetyl on peracetylated uridine and cytidine was achieved in good yields without observing acetyl transfer (89 % vs 77 %, while the *N*‐acetyl group was also cleaved for tetraacetylcytidine). The organotin most likely served as a mild Lewis acid in the transesterification process, but evidence also indicated a mechanism for which the Lewis acidity is not of primary importance.^[46]^ Other organotin reagents such as dibutyltin oxide (DBTO) was also employed for chemoselective de‐*O*‐acetylation in a chloropurine nucleoside derivative; however, the selectivity was uncertain and a mixture of products were obtained.[^48^, ^49^]

Another chemical approach involved heating peracetylated nucleosides in a 1 % (w/v) iodine‐methanol solution.^[50]^ The use of readily available and relatively inexpensive reagents, in addition to an easy reaction workup, make this method an attractive option for selective deacetylation. Regioselective deprotection of the primary acetyl group on five peracetylated natural nucleosides occurred in moderate to good yields (55–69 %, 0.1–1 g scale). However, for tetraacetylcytidine, only the *N* ^4^‐acetyl group was removed under heating, indicating that the exocyclic amide carbonyl carbon is more electrophilic than the esters are.^[51]^ Presumably, the selective methanolysis mechanism involves the coordination of an electrophilic iodine species[^50^, ^52^] with the carbonyl oxygen of the most sterically accessible primary acetyl group. The electrophilic iodine was partly evidenced by the formation of a small amount of 5‐iodo‐2′,3′,5′‐tri‐*O*‐acetylcytidine when tetraacetylcytidine was treated with the reagent. Some acetylated glucose derivatives also underwent selective deprotection at the 6‐OH.^[50]^

While there are few chemical methods for selective deacetylation on primary acetyl esters in nucleosides, a number of enzymatic methods have been reported (Table 1). The use of different lipases or esterases is common, but some proteases and whole microorganisms have also proved to be effective. Among these enzymes, the most well‐studied and widely used is *Candida antarctica* lipase B (CALB), a lipase with well‐documented high activity on a wide variety of non‐natural substrates.^[53]^

**Table 1:** Biocatalytic methods for selective de‐*O*‐acetylation on primary esters in nucleosides.

Enzyme	Substrate(s)	Yield (Scale)	Notes	Ref.
Novozyme‐435 (CALB)	2′,3′,5′‐Tri*‐O‐*acetyluridine	92 %^#^ (200 mg)	Analyzed the role of the alcohol/substrate molar ratio, alcohol/biocatalyst and substrate/biocatalyst mass ratios for the selective ethanolysis of peracetylated uridine.	[54]
	*N*7/*N*9 regioisomers of β‐D‐ribofuranosylguanines, α‐D‐arabinofuranosylguanines, and α‐L‐arabinofuranosylguanines	72 % for *N*9 isomer* (200 mg)	The enzyme only regioselectively deacetylated the *N*9 isomers to achieve separation of the mixtures.	[58]
	Triacetyluridine, and triacetyl‐2′‐methyluridine	71–93 %^#^ (1 mmol)	In biocatalytic alcoholysis with n‐butanol, peracetylated uridine had a lower yield than 2′‐methyluridine; in selective hydrolysis, deacetylation only succeeded on peracetylated 2′‐methyluridine.	[60]
	4′‐C‐acyloxymethyl‐2′,3′,5′‐tri‐ *O*‐acyl‐β‐D‐xylofuranosyl nucleosides	82–93 %* (1.09 mmol)	n‐Butanol was added in THF (solvent) as the acetyl acceptor. This enzyme solely removed the acyl group from the C4′‐acyloxymethyl function for substrates, producing the key intermediate for the synthesis of xylo‐LNA monomers.	[56]
	2′,3′,5′‐Tri‐*O*‐acetyl‐1‐β‐D‐arabino‐ furanosyluracil and 2′,3′,5′‐tri‐*O*‐acetyl‐9‐β‐D‐arabino‐ furanosyladenine	72 %^#^ (U) 83 %^#^ (A) (0.20 mmol)	Hydrolysis of the substrates in phosphate buffer afforded the 2′,3′‐di‐*O*‐acetylated arabino‐ nucleosides while alcoholysis with isopropanol gave 2′‐*O*‐acetylated products.	[65]
CALB CLEA	5‐Fluoro‐*N* ^4^‐(n‐pentyloxycarbonyl)‐ 2′,3′,5′‐tri*‐O*‐acetylcytidine	82 %^#^	Enzyme cross linked aggregate (CLEA Technologies) of CALB was used. Enzyme‐catalyzed alcoholysis of the triacetate substrate in ethanol mainly afforded the 5′‐ hydroxy‐2′,3′‐diacetate, at 93 % conversion.	[67]
*Burkholderia cepacia* esterase (SC esterase S)	Tetraacetylcytidine	95 %* (1000 mg)	*Aspergillus niger* lipase (Amano A) was also tested and the substrate tetraacetylcytidine was fully de‐*O*‐acetylated to afford *N* ^4^‐acetylcytidine (77 %).	[68]
Acetyl esterase (AE) from the flavedo of oranges	*N*‐phenylacetyl‐3′,5′‐di*‐O*‐acetyl‐ 2′‐deoxyadenosine, and *N*‐phenylacetyl‐3′,5′‐di*‐O*‐acetyl‐ 2′‐deoxyguanosine		For nucleosides with protected amino groups, the primary 5′‐OH groups were liberated. However, for substrates that do not carry *N*‐protecting groups, the selectivity was “reversed” and deacetylation happened at the secondary 3′ position.	[71]
Subtilisin	Various peracetylated nucleosides^[a]^	40–92 %* (250 mg)	Protease N and lipase from porcine pancreas (PPL) were also screened using 2′,3′,5′‐tri‐*O*‐ acetyluridine. Protease N (10 times more enzyme needed) showed good selective deacetylation activity but produced slightly lower yields than subtilisin‐catalyzed reactions.	[73]
Subtilisin (Alcalase CLEA)	5‐Fluoro‐*N* ^4^‐(n‐pentyloxycarbonyl)‐ 2′,3′,5′‐tri*‐O*‐acetylcytidine	91 %^#^ 80 %* (1.72 g)	A cross‐linked enzyme aggregate (CLEA) subtilisin catalyzed alcoholysis of the substrate afforded the 5′‐hydroxyderivative at 96 % conversion. Common commercially available lipases were tested but showed low selectivity.	[67]
Whole microbial cells of *Cellulomona celulans* & *Klebsiella sp*.	2′,3′,5′‐Tri‐*O*‐acetyladenosine and 2′,3′,5′‐tri‐*O*‐acetyluridine^[b]^	>70 %^#^ (1 mM/3 mL)	Various nucleosides and microorganisms were tested.^[b]^ However, regioselective deacetylation could only be achieved with 2′,3′,5′‐tri‐*O*‐acetyl‐ adenosine and 2′,3′,5′‐tri‐*O*‐acetyluridine as substrates and with *Cellulomona celulans* and *Klebsiella sp*. as biocatalysts.	[76]

* Isolated yield. ^#^ Determined by HPLC. [a] Nucleosides used were 2′,3′,5′‐tri‐*O*‐acetyluridine, 2′,3′,5′‐tri*‐O*‐acetylcytidine, 2′,3′,5′‐tri‐*O*‐acetyladenosine, 2′,3′,5′‐tri‐*O*‐acetylinosine, 2′,3′,5′‐tri*‐O*‐acetylguanosine, and 2‐*N*‐acetyl‐2′,3′,5‐tri‐*O*‐acetylguanosine. [b] Nucleosides tested were 2′,3′,5′‐tri‐*O*‐acetyladenosine, 2′,3′,5′‐tri‐*O*‐acetyluridine, 2′,3′,5′‐tri*O*acetylinosine, 2′,3′,5′‐tri‐*O*‐acetylcytidine, 4‐*N*‐acetyl‐2′,3′,5′‐tri*‐O*‐acetylcytidine, 2′,3′,5‐tri‐*O*‐acetylguanosine, and 2‐*N*‐acetyl‐2′,3′,5‐tri‐*O*‐acetylguanosine.

**Scheme 2:**
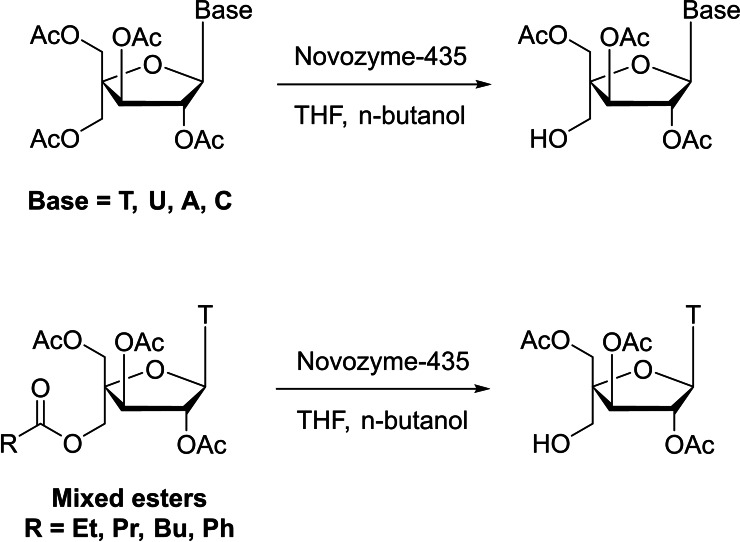
Novozyme‐435 solely removes the acyl group from the C4′‐acyloxymethyl function in these tetra‐*O‐*acetylated *xylo*furanosyl nucleosides.

CALB, particularly Novozyme‐435 (the commercial biocatalyst with immobilized CALB),^[54]^ has been found to be very effective for de‐*O‐*acetylation on primary esters.^[55]^ The reaction also usually involves an easy work up, since the enzyme is an immobilized biocatalyst. For example, treating complex tetra‐*O‐*acetylated 4′‐C‐hydroxymethyl *xylo*furanosyl nucleosides with Novozyme‐435, using n‐butanol as an acyl acceptor, produced high yields (82–93 %) of monodeacetylated 4′‐hydroxymethyl products on a sub‐gram scale (Scheme 2).^[56]^ Importantly, when mixed esters were used, the biocatalytic deacylation reaction gave the same monodeacylated nucleosides (Scheme 2).^[56]^ These studies clearly indicated that this enzyme solely acted on the removal of the acyl group from the primary C4′‐acyloxymethyl function for these substrates and ruled out the possibility of acyl migration during the reaction. Other lipases were tested and did not show any selectivity for such substrate. The reaction afforded the key intermediate for the synthesis of xylo‐LNA monomers for modified oligonucleotides.^[57]^

**Scheme 3:**
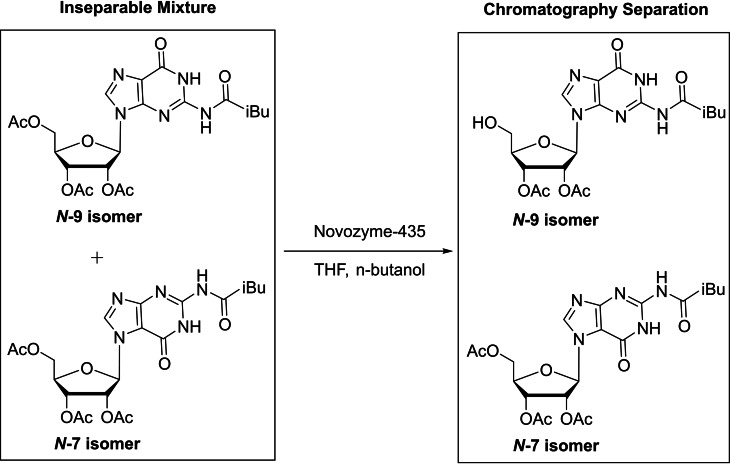
Novozyme‐435 solely hydrolyzes the primary acetyl group of peracetylated *N*‐9 guanosine isomers, enabling complete chromatographic separation.

Novozyme‐435 was also used to separate mixtures of *N*‐7/*N*‐9 guanine nucleosides, including β‐D‐ribofuranosylguanines, α‐D‐arabinofuranosylguanines, and α‐L‐arabinofuranosylguanines.^[58]^ These nucleosides were synthesized with peracetylated sugars and contained *N*‐7/*N*‐9 regioisomers that are almost impossible to separate by chromatographic techniques. The enzyme selectively hydrolyzed the primary acetyl group at the C‐5′ position of tri‐*O*‐acetylated *N*‐9 isomers, enabling the separation of mixtures of *N*‐7 and *N*‐9 isomers with silica gel column chromatography (Scheme 3).^[58]^

In another report, Novozyme‐435^[59]^ was studied for lipase‐catalyzed alcoholysis and hydrolysis of peracetylated uridine and 2′‐methyluridine.^[60]^ It was discovered that, in the enzymatic alcoholysis, the ethanol/nucleoside ratio (E/N) had a significant impact on the results. In a very high excess of ethanol (E/N >1000) selective deacetylation of the primary acetate was observed to afford 2′,3′‐di‐*O*‐acetyl uridines (71–93 % yield on a 1 mmol scale, and peracetylated uridine had a lower yield than 2’‐methyluridine).[^60^, ^61^, ^62^] However, under hydrolysis conditions in potassium phosphate buffer, regioselective deacetylation only worked on peracetylated 2′‐methyluridine (61 %–100 % yield, depending on temperature). This study also tested the effectiveness of *Candida rugosa* lipase from *Candida cylindracea* (CRL), porcine pancreatic lipase (PPL), and lipase from *Rhisomucor miehei* (LIP) in the alcoholysis, but no deacetylation reaction of peracetylated uridine was observed using these enzymes.^[60]^ Later, another group studied the reaction details of the Novozyme‐435 catalyzed alcoholysis of peracetylated uridine in an attempt to explain the exclusive/selective 5′ de‐*O*‐acetylation at very high alcohol amounts.^[54]^ They investigated the ethanol/substrate molar ratio, and ethanol/biocatalyst and substrate/biocatalyst mass ratios. Under optimal conditions, a conversion rate of 92 % could be achieved on a 200‐mg scale.^[54]^ Based on their experimental and theoretical analysis, it was concluded that a potential allosteric effect of ethanol,[^63^, ^64^] stronger in combination with water, resulted in the high selectivity towards 5′ de‐*O*‐acetylation.

Interestingly, different results/types of selectivity were observed when two peracetylated arabinonucleosides were treated with CALB in alcoholysis vs. hydrolysis (Table 1).^[65]^ Hydrolysis of the substrates in phosphate buffer afforded the 2′,3′‐di‐*O*‐acetylated arabinonucleosides while alcoholysis with isopropanol surprisingly gave 2′‐*O*‐acetylated products (3′,5′‐di‐*O*‐deacetylation).^[65]^ The different results were probably due to the influence of the arabinofuranose moiety on CALB recognition, and this procedure allows a simple preparation of the key intermediate for the synthesis of vidarabine prodrugs.^[66]^ For some synthesized non‐natural nucleoside substrates, a special commercially processed CALB was also explored – CALB CLEA (crossed linked enzyme aggregate by CLEA technologies).^[67]^ These aggregates possess superior properties such as improved stability, activity, and tolerance of organic solvents. For example, CALB CLEA catalyzed selective alcoholysis of non‐natural 5‐fluoro‐*N* ^4^‐(n‐pentyloxycarbonyl)‐2′,3′,5′‐tri*‐O*‐acetylcytidine, providing the nucleoside with a free 5′‐OH group in great yield and conversion.^[67]^

**Scheme 4:**
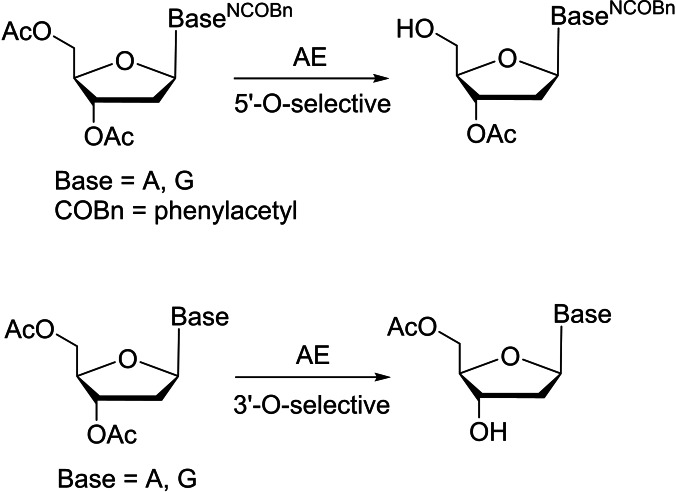
Acetylesterase (AE) from orange peel showed distinct selectivity on 2‐deoxy purine nucleosides (NCOBn=purine base exocyclic amino group protected with phenylacetyl group).

While the CALB lipase is being extensively studied for such transformation, other types of enzymes, such as esterases, have also demonstrated catalytic activity and selectivity on the primary ester of acetylated nucleosides. For example, SC esterase S (*Burkholderia cepacia* esterase) was utilized to selectively deprotect the 5′‐*O*‐acetyl on tetraacetylcytidine. The triacetylated product 2′,3′‐*O*,*N* ^4^‐triacetylcytidine was afforded in excellent yield (95 %) on a 1 gram scale. Unfortunately, they did not further investigate the substrate specificity of this enzyme on other peracetylated nucleosides.^[68]^ In addition, the enzyme acetyl esterase (AE) from orange peel has been used in regioselective deprotection of carbohydrates and nucleotides.[^69^, ^70^] Waldmann and coworkers discovered that AE demonstrated distinct selectivity in deacetylating 2‐deoxy purine nucleosides (Scheme 4).^[71]^ For nucleosides with phenylacetyl (COBn) protected exocyclic amino groups, the primary 5′‐OH groups were liberated. Conversely, for substrates that did not carry *N*‐protecting groups, the selectivity was reversed and deacetylation occurred at the secondary 3′ position.[^71^, ^72^] However, for such 3′‐*O*‐deacetylation, it was not determined whether alternatively the primary acetate was cleaved first followed by a 3′→5′ acetyl migration blocking the primary hydroxy group.

In addition to lipases and esterases, proteases were also explored and showed promising activity in regioselective deprotection of peracetylated nucleosides.[^72^, ^73^] In a pioneer research, Singh *et al*. discovered that the protease subtilisin could regioselectively hydrolyze 2′,3′,5′‐tri‐*O*‐acetyluridine with almost quantitative transformation (100 % yield by HPLC). The crude subtilisin (protease N; Amano) was also tested; however, 10 times more enzyme was required to achieve a similar yet slightly lower yield.[^72^, ^73^] Subsequently, they screened six common peracetylated nucleosides (see Table 1 footnote) using subtilisin on a 250 mg scale, and moderate to excellent isolated yields (40–92 %) were obtained. This demonstrated the broad substrate specificity of this enzyme and great potential in the synthesis of important pharmaceutical intermediates. Based on this work, another group reported the use of the cross‐linked enzyme aggregate of subtilisin (Alcalase CLEA) to carry out regioselective ethanolysis of a complex cytidine derivative (5‐fluoro‐*N* ^4^‐(n‐pentyloxycarbonyl)‐2′,3′,5′‐tri‐*O*‐acetylcytidine).^[67]^ In a gram‐scale synthesis, the catalysis afforded the acetylated derivative with a free 5′‐hydroxy in 80 % isolated yield. Common lipases were also examined but were less effective on this substrate. The 5′‐hydroxyderivative is a crucial intermediate for the synthesis of antitumoral capecitabine (brand name Xeloda^®^), which was developed as a prodrug of fluorouracil but proved to be more efficacious and significantly less toxic.[^74^, ^75^]

The above‐mentioned selective de‐*O*‐acetylation on the 5′ position using isolated enzymes (including lipases, esterases, and proteases) are summarized in Table 1. Enzymatic reactions are controllable, scalable, and usually occur under mild conditions. However, purified or processed enzymes are required, which can limit their applications due to availability and/or increased synthesis cost. On the other hand, transformation using whole cells of microorganisms has been explored as a promising alternative.^[76]^ Iribarren group pioneered in the use of biotransformation in nucleoside chemistry, such as the synthesis of nucleosides through biocatalysis employing microbial whole cells.[^77^, ^78^] In their recent work, they screened microbial cells for selective hydrolysis of a set of peracetylated nucleosides (Table 1), aiming to identify new sources of hydrolases.^[76]^ Various microorganisms were tested, but regioselective deacetylation to afford the 2′,3′‐di‐*O*‐acetylnucleosides could only be achieved for adenosine and uridine, with *Cellulomona celulans* and *Klebsiella sp*. as biocatalysts, respectively. It was also determined that, unlike enzyme‐catalyzed reactions, none of the microorganisms that were used displayed a common reaction profile for deacetylation; some microorganisms gave mixtures of different diacetylated products while others afforded a mixture of monoacetylated products.

## 3 Selective De‐*O*‐Acetylation ‐ Secondary Ester

**Scheme 5:**
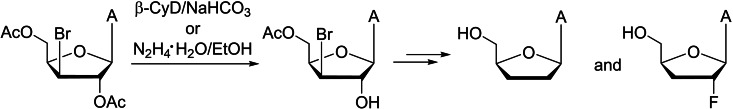
Regioselective 2′‐*O‐*deacetylation with β‐cyclodextrin in *aq*. NaHCO_3_ or N_2_H_4_ ⋅ H_2_O.

Selective de‐*O*‐acetylation of nucleosides typically occurs on the primary *O*5′ position, since it is the most sterically accessible position. However, numerous studies have successfully removed one or both of the secondary esters on peracetylated nucleosides while keeping the primary acetyl intact. In an early research, Shiragami *et al*. achieved the regioselective 2′‐*O‐*deacetylation of 9‐(2′,5′‐di‐*O‐*acetyl‐3′‐bromo‐3′‐deoxy‐β‐D‐xylofuranosyl)adenine by treating the derivative with either β‐cyclodextrin (β‐CyD) in *aq*. NaHCO_3_ or N_2_H_4_ ⋅ H_2_O in ethanol, which led to the synthesis of a few 2′,3′‐dideoxynucleosides as potent anti‐HIV agents (Scheme 5).^[79]^ β‐CyD may have formed a certain complex with the substrate as revealed by ^1^H NMR experiment, and no such effect was observed when α‐CyD was used.^[80]^ It is also worth noting that 5′‐*O‐*deacetylation product was formed predominantly if treated with aqueous HCl. However, the HCl concentration/condition and % yield were not reported.

**Scheme 6:**
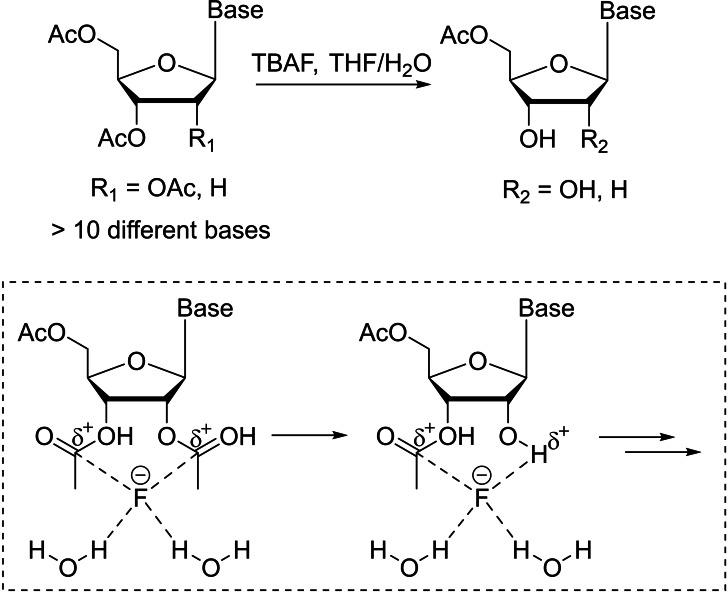
Regioselective *O*2′,*O*3′‐deactylation of peracetylated nucleosides with TBAF and the proposed mechanistic pathway.

Notably, Manetsch and coworkers reported a novel chemical approach to selectively deacetylate the *O*2′, and *O*3′‐positions using tetra‐*n*‐butylammonium fluoride (TBAF) without disturbing the *O*5′ acetyl group (Scheme 6).^[81]^ A wide variety of peracetylated ribonucleosides and derivatives were examined, and good to excellent yields were obtained (62–90 %), evidencing the broad scope and robustness of this method. In particular, substrates with susceptible functionality on nucleobases such as 8‐bromoadenine and *N* ^4^‐acetylcytosine were all well‐tolerated under the reaction conditions. This research also demonstrated a promising route for selective *O*3′‐deacetylation of peracetylated 2′‐deoxyribonucleosides, although the selectivity was not as high as it was for the peracetylated ribonucleosides, and only moderate yields were observed (53–55 %) (Scheme 6). Further studies revealed that the tetra‐n‐butylammonium and fluoride ion pair were crucial, which led to the deduction of the following reaction mechanism: (1) poor binding ability of tetra‐n‐butylammonium allows the fluoride ion to freely coordinate to the electron‐deficient carbonyl carbon^[82]^ atoms attached to the *O*2′‐ and *O*3′‐positions; (2) nucleophilic attack of the coordinated fluoride on the carbonyl carbon to remove the acetyl group; and (3) the cycle of coordination and deacetylation continues to give the *O*2′,*O*3′‐deactylation product. This mechanism also explained the origin of selectivity, as deacetylation occurs only if the acetate group is in close proximity, i. e. cyclic 1,2‐diol diacetate, for fluoride ion coordination (Scheme 6). This mechanism can be further exploited for easy access to 1,2‐diols in carbohydrate and natural product synthesis. The obtained *O*5′‐acetyl ribonucleosides are important intermediates for the synthesis of biochemically important molecules such as oligonucleotides and deoxynucleosides.[^83^, ^84^]

**Scheme 7:**
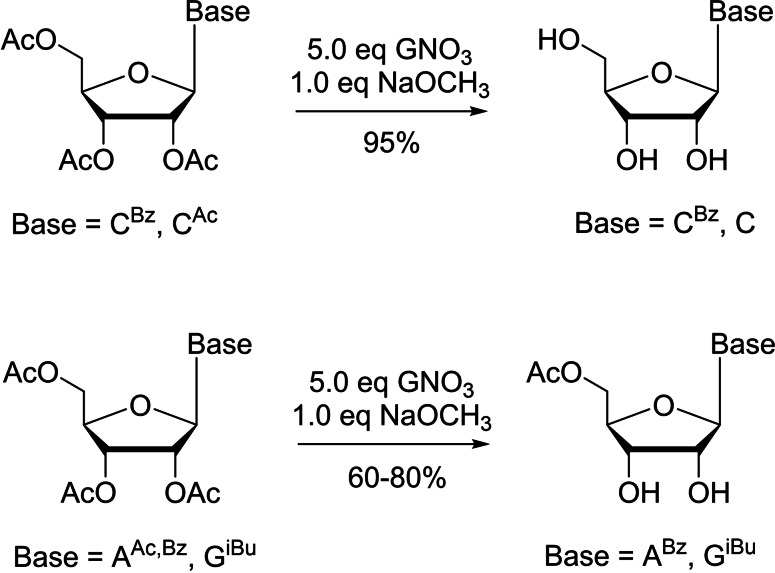
Selective 2′,3′‐di‐*O*‐deacetylation of peracylated purine (A, G) nucleosides using guanidinium nitrate (GNO_3_)/sodium methoxide reagent mixture.

Additionally, in a study using a guanidinium nitrate (GNO_3_)/sodium methoxide reagent mixture to achieve de‐*O*‐acylation of peracylated ribonucleosides (with *N*‐acylated nucleobases), a special selectivity was observed in which 2′,3′‐di‐*O*‐deacetylated products were exclusively formed from peracylated purine nucleosides (Scheme 7).^[85]^ Another research demonstrated that the use of a well‐defined concentration of ammonia and a specific reaction temperature could result in selective deacetylation and debenzoylation on the secondary hydroxy groups in some nucleosides and carbohydrates.^[86]^ More applications of these simple reagents are certainly worth investigating in nucleoside and carbohydrate chemistry.^[87]^ The aforementioned lipase CALB was also explored to afford products bearing different degrees of acetylation with different reaction media in the deacetylation of 2′,3′,5′‐tri‐*O*‐acetyl‐1‐β‐D‐arabinofuranosyluracil and 2′,3′,5′‐tri‐*O*‐acetyl‐9‐β‐D‐arabinofuranosyladenine.^[65]^ In isopropanol, CALB catalyzed alcoholysis resulted in selective 3′,5′‐di‐*O*‐deacetylation in excellent yields (Table 1).^[65]^ Full deacetylation of the substrates occurred when using a set of commercial hydrolases and fungal keratinases.

## 4 De‐*O‐*Acetylation of *N*,*O*‐Acetylated Nucleosides and De‐*N*‐Acetylation

**Scheme 8:**
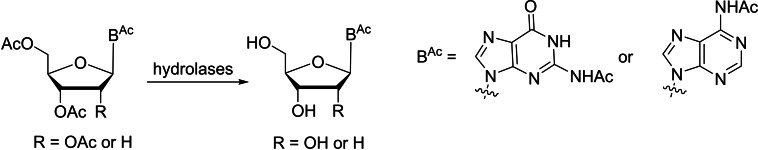
*N*‐acetyl protected purine nucleosides from chemoselective enzymatic hydrolysis.

Other forms of regioselective deacetylation, such as full de‐*O‐*acetylation of *N*,*O*‐acetylated nucleosides and selective de‐*N‐*acetylation, can be realized by taking advantage of the reactivity difference between ester and amide bonds. For example, various lipases could be used to catalyze the full de‐*O‐*acetylation of *N*,*O*‐acetylated nucleosides.^[88]^ As mentioned before (Table 1), the substrate tetraacetylcytidine was fully de‐*O*‐acetylated, using *Aspergillus niger* lipase (Amano A), to afford *N* ^4^‐acetylcytidine on a 900 mg scale (77 % isolated yield).^[68]^ In another catalysis study, *N*‐acetyl protected ribo‐ and 2′‐deoxyribopurine nucleosides were afforded from the corresponding *N*,*O*‐peracetylated compounds using the following four enzymes (Scheme 8): acylase I (*Aspergillus melleus*), Novozyme‐435 (CALB), esterase from pig liver (PLE), and grade II from porcine kidney (PKA‐II). Both the acylase I and Novozyme‐435 hydrolyzed all four substrates in 79–100 % conversion.^[89]^ The esterase from pig liver and grade II from porcine kidney were more effective on *N*,*O*‐peracetylated guanosine and 2′‐deoxyguanosine, respectively, and de‐*O*‐acetylations were almost quantitative. This study supported the assumption that lipase CALB and esterase PLE catalyze ester, but not amide, hydrolysis, although some previous works had reported that both lipases and esterases catalyzed amide hydrolysis.[^89^, ^90^, ^91^, ^92^] In addition, the above‐mentioned keratinases, produced by some insects and mainly by microorganisms,^[93]^ could also be further explored for full de‐*O‐*acetylation.

**Scheme 9:**
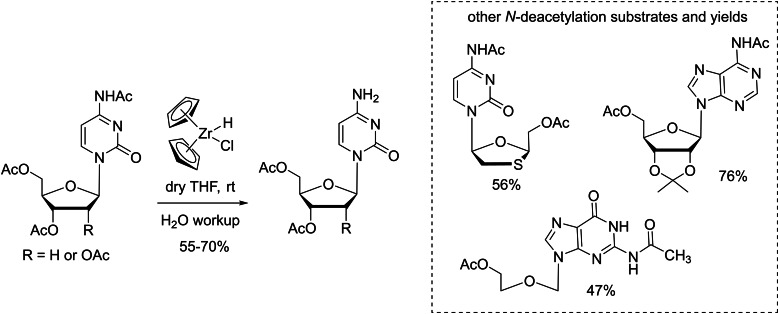
Selective de‐*N‐*acetylation of protected nucleosides and analogues promoted by *Schwartz's* reagent.

On the other hand, protecting and deprotecting the *N*‐acetyl group are equally important and widely used in nucleoside and nucleotide chemistry. Generally, de‐*N*‐acetylation requires harsh acidic or basic conditions in which other protection groups such as esters would be highly labile. Pertusati and coworkers reported a mild chemical strategy using an organozirconium compound (known as *Schwartz's* reagent) to regioselectively remove the *N*‐acetyl group on nucleobases.^[94]^ Peracetylated pyrimidine and purine nucleosides and some analogues were tested, including *O,N*‐diacetylated Lamivudine, *N*‐acetyl‐5‐*O*‐acetyl‐2,3‐*O*‐isopropylidene adenosine and peracetylated acyclovir, and de‐*N*‐acetylation products were obtained in good yields (Scheme 9). More importantly, this reagent/method could tolerate a wide variety of protecting groups, such as acyl groups, ether, silyl ether, acetal, and *tert*‐butoxycarbony (Boc) group, as the reagent is highly preferential to amides via a reductive cleavage mechanism.[^95^, ^96^, ^97^] It is also compatible with *N*‐acetylated cytidine containing a phosphoramidate motif, which serves as an innovative nucleoside monophosphate prodrug.[^94^, ^98^, ^99^]

Another method demonstrated that solutions of *N,O*‐peracetylated adenosine and cytidine and their derivatives undergo selective de‐*N*‐acetylation in superheated methanol (105 °C).^[100]^ Amides are usually more stable/less reactive because nitrogen is an effective donor of electrons to the carbonyl group. However, in this case, a shift of electron density from the exocyclic amino *N* into the π‐deficient nucleobase ring causes the amide carbonyl carbon to become more electrophilic, allowing selective de‐*N*‐acetylation in the presence of more stable esters.^[100]^ Reaction time was shorter and yield higher for adenosine (1 h, 84 %) than cytidine (3 h, 61 %), evidencing that *N* ^6^ amides of adenosine are more electrophilic and reactive than those derived from cytidine. Other *O*‐protecting groups (acyl, sulfonyl, TBS) were compatible, and increasing the bulk of the *N*‐acyl group increased the *N/O* selectivity ratio by decreasing the rate of de‐*O*‐acylation.

## 5 Summary and Outlook

Nucleosides and nucleoside analogues are key building blocks for the synthesis of various types of RNAs and DNAs and for use as antiviral drugs and anticancer and cardioprotective agents. Nucleosides have multiple reactive sites, and thus regioselective protection and deprotection of specific hydroxy groups (or amino groups) is crucial and represents one of the major challenges in nucleoside modification. Esters are of fundamental importance in nucleoside and carbohydrate chemistry and the acetyl group is one of the most widely used protecting groups in synthetic work due to the high efficiency of acetylation reactions. Thus, developing regioselective deacetylation methods will greatly simplify the preparation of partially or differentially substituted nucleoside intermediates for structural modification.[^36^, ^37^] In this review, we summarized and evaluated methods for the selective deacetylation of peracetylated nucleosides, which includes approaches for the regioselective de‐*O‐*acetylation of primary esters and secondary esters, full de‐*O*‐acetylation of *N,O*‐acetylated nucleosides, and selective de‐*N‐*acetylation of nucleosides that contain an exocyclic amide. Both chemical and enzymatic approaches were surveyed, and removal of the acetyl group relies on either alcoholysis or hydrolysis in both methods.

Compared to chemical approaches, enzymatic methods are more widespread for the selective deacetylation of nucleosides, and are thus more likely to be used in the biochemical field. Biocatalysis often offers advantages over chemical synthesis because enzyme‐catalyzed reactions are usually performed under mild conditions and are environmentally friendly. Because of their substrate specificity, enzymes were also used to hydrolyze a particular regioisomer from the inseparable isomeric mixture to achieve separation.^[58]^ Meanwhile, a number of enzymes have been used on various substrates (natural and analogues) and their substrate specificity could be further investigated. The CALB lipase, in particular, possesses a very relaxed substrate specificity that includes nucleosides, D‐ribonolactones,^[101]^ and other sugars, and demonstrates high performance for each of these substrates. By contrast, chemoselective approaches, though less common, take advantage of a unique structural motif and do not require specialized and/or expensive enzymes. Therefore, they can be more universal and are not limited to nucleosides or carbohydrates. Chemical methods also exhibit overall faster reactions (0.5–40 hours) compared to enzymatic selective deacetylation (4–168 hours). They can be performed on a larger scale and involve simpler product isolation and purification processes. Overall, both methods have great potential to be further explored and extended to a broader scope in carbohydrate chemistry.[^27^, ^87^, ^102^] We hope this review intrigues and inspires chemists and biochemists, leading to an increased availability of new systems and robust biocatalysts.

## Conflict of Interests

The authors declare no conflict of interest.

## Biographical Information

*Dr. Cai was born in Chengdu, China. He received his B.S. degree in Pharmaceutical Sciences and M.S. degree in Medicinal Chemistry from Peking University. He then attended The Ohio State University, where he earned his Ph.D. in Organic Chemistry. Dr. Li Cai joined the faculty of the University of South Carolina in 2011 and is now a Professor of Chemistry at USC Lancaster. He is an organic chemist and glyco‐scientist whose research focuses on carbohydrate chemistry, enzymatic synthesis of carbohydrates, and chemical glycobiology. He is also devoted to enhancing the teaching and learning of chemistry, which includes advising undergraduate researchers*.

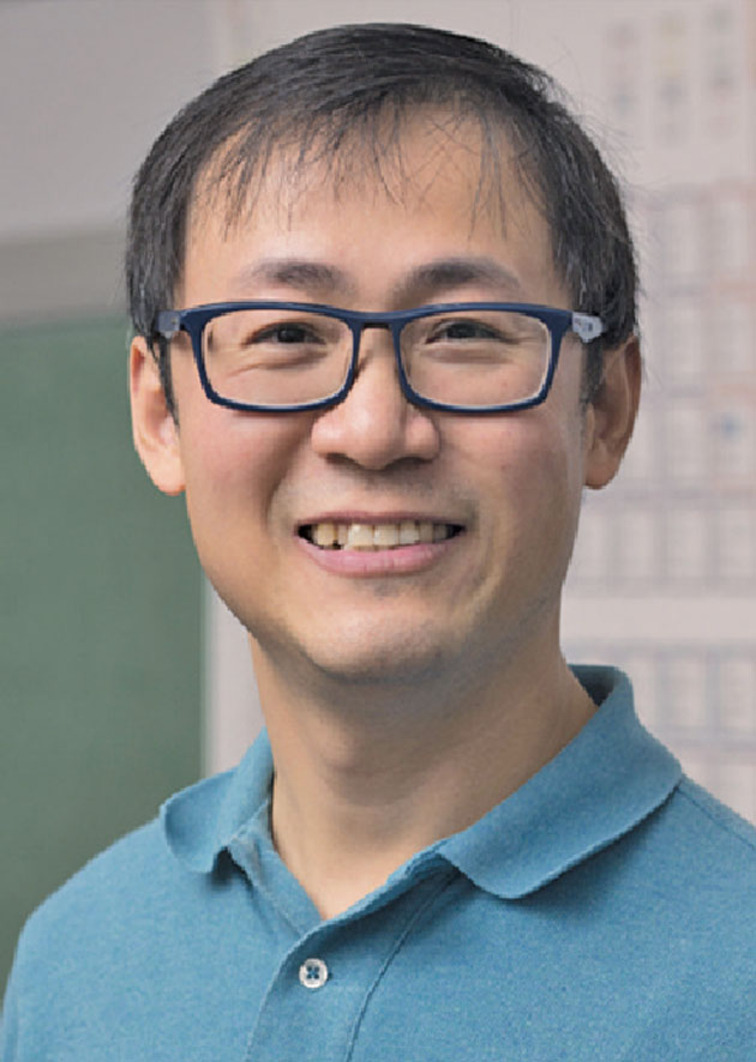

## Biographical Information

*Ms. Charis Grabbe is a junior at USC Palmetto College, where she is pursuing a Bachelor of Liberal Studies degree in Biology and Chemistry with a cognate in Business. She has done extensive research with a focus on VOCs in floral scent, and recently began exploring nucleoside chemistry. She served as president of the USCL Chemistry Club and worked as a chemistry lab assistant on campus. Currently, she is an intern in the CSI lab at the Lancaster County Sheriff's office. Charis hopes to get her Master Gardener and Master Naturalist certifications after graduation and become an Outdoor Educator*.